# Facial Expression Realization of Humanoid Robot Head and Strain-Based Anthropomorphic Evaluation of Robot Facial Expressions

**DOI:** 10.3390/biomimetics9030122

**Published:** 2024-02-20

**Authors:** Zhibin Yan, Yi Song, Rui Zhou, Liuwei Wang, Zhiliang Wang, Zhendong Dai

**Affiliations:** 1 College of Mechanical and Electrical Engineering, Nanjing University of Aeronautics and Astronautics, Nanjing 210016, China; zhibinyan@nuaa.edu.cn (Z.Y.); zhourui@nuaa.edu.cn (R.Z.); wangliuwei@nuaa.edu.cn (L.W.); 2 School of Mechanical Engineering, Zhejiang University of Technology, Hangzhou 310014, China; yisong@zjut.edu.cn; 3 School of Computer and Communication Engineering, University of Science and Technology Beijing, Beijing 100083, China; wzl@ustb.edu.cn; 4 Institute of Bio-Inspired Structure and Surface Engineering, Nanjing University of Aeronautics and Astronautics, Nanjing 210016, China

**Keywords:** vertical graphene sensor (VG Sensor), strain measurement, facial expression evaluation, humanoid robot head

## Abstract

The facial expressions of humanoid robots play a crucial role in human–computer information interactions. However, there is a lack of quantitative evaluation methods for the anthropomorphism of robot facial expressions. In this study, we designed and manufactured a humanoid robot head that was capable of successfully realizing six basic facial expressions. The driving force behind the mechanism was efficiently transmitted to the silicone skin through a rigid linkage drive and snap button connection, which improves both the driving efficiency and the lifespan of the silicone skin. We used human facial expressions as a basis for simulating and acquiring the movement parameters. Subsequently, we designed a control system for the humanoid robot head in order to achieve these facial expressions. Moreover, we used a flexible vertical graphene sensor to measure strain on both the human face and the silicone skin of the humanoid robot head. We then proposed a method to evaluate the anthropomorphic degree of the robot’s facial expressions by using the difference rate of strain. The feasibility of this method was confirmed through experiments in facial expression recognition. The evaluation results indicated a high degree of anthropomorphism for the six basic facial expressions which were achieved by the humanoid robot head. Moreover, this study also investigates factors affecting the reproduction of expressions. Finally, the impulse was calculated based on the strain curves of the energy consumption of the humanoid robot head to complete different facial expressions. This offers a reference for fellow researchers when designing humanoid robot heads, based on energy consumption ratios. To conclude, this paper offers data references for optimizing the mechanisms and selecting the drive components of the humanoid robot head. This was realized by considering the anthropomorphic degree and energy consumption of each part. Additionally, a new method for evaluating robot facial expressions is proposed.

## 1. Introduction

Facial expressions and body language are crucial influencing factors in interpersonal communication, constituting 55% of interpersonal communication information [1,2,3]. With the rapid development of humanoid robots, such as Tesla Optimus Prime, it is imperative to investigate methods for improving the anthropomorphism of robot facial expressions and to propose evaluation methods for robot facial expressions. This approach will guide the study of robot facial expressions toward a more scientific trajectory [4,5]. Over the past two decades, significant progress has been made regarding the research of facial expressions and humanoid robot heads. Notably, robots without skin include WE-4RII [6,7] series robots, KOBIAN [8] humanoid robots, “SHFR-III” [9,10] human head robots, Kismet [11] head robots, and Nexi [12] robots developed by MIT. Additionally, Disney has developed a skinless robot with a gaze feature [13]. Robots without skin can universally achieve mechanical facial features through mechanisms, which makes it easier to avoid the ‘Uncanny Valley’. Robot behavior generation aims to emulate humanoid behavior. Facial expressions achieved by humanoid robot heads with skin are more anthropomorphic and hold greater research significance. Currently, humanoid robot heads with skin [14], such as SAYA [15,16] and Sofia [17], use stepping motors, cylinders or McKibben pneumatic actuators to achieve facial expressions. Although these robots can achieve diverse facial expressions and incorporate voice communication, they have not yet gained popularity due to their heavy weight, high cost, and limited load-carrying capacity. Moreover, the H&Frobot-Ⅲ [18] human head robot, which is the human head robot developed by Yu et al. [19], the artificial emotion robot developed by Wang et al. [20,21], the human robot head developed by Tadesse et al. [22,23] and the “Eva” robot face machine [24,25], among others, have used a rope mechanism or a rope-linkage-combined mechanism to drive the robot’s skin. However, this driving method is plagued by a complex structure, easy to move interference, low control accuracy, and actuator relaxation. Lin et al. [26,27] developed a humanoid robot head with a limited number of actuators, thus overcoming the problem of mechanical complexity, but it was limited to a fixed number of facial expressions. Numerous social robots that are capable of facial expressions have been developed [28], but there is no quantitative method to evaluate the degree of anthropomorphism in robot facial expressions. Presently, the anthropomorphic degree of robot facial expressions is primarily evaluated by subject recognition experiments [29]. However, significant differences between subjects, regarding the ability to recognize robot facial expressions, can lead to less accurate assessment results. Therefore, proposing an evaluation method to ascertain the degree of anthropomorphism in robot facial expressions holds great significance. The robot’s facial expression is achieved by skin deformation. Thus, the distribution of motion control points on the skin, skin strain, and stress, as well as the skin material, are all crucial factors that influence the robot’s ability to achieve facial expression. Presently, there are few studies on the strain and stress of the skin when the robot completes the facial expression. Ke et al. [30] conducted finite element analysis on the robot’s facial expression, they obtained the basic facial expression simulation diagram, and the optimal driving load for completing the facial expression. However, there is a lack of experimental measurements. Misu et al. [31] estimated the area strain distributions from discrete measurement points on the face, but this depends on several approximations and assumptions.

Therefore, based on FACS, this paper designed and manufactured a 19-degrees-of-freedom humanoid robot head with skin. To improve the response speed, transmission efficiency, and load-bearing capacity of the humanoid robot head, the servo drive was utilized. The driving force of the mechanism was efficiently transmitted to the silicone skin through a rigid linkage drive and snap button connection, this snap button connection method was implemented to improve the lifespan of the silicone skin. Secondly, Maya 2020 simulation software was employed to simulate six basic facial expressions, based on human facial expressions, and to obtain relevant motion parameters. Subsequently, the robot’s control system was designed to achieve these facial expressions. We determined the measurement sites based on the distribution of motion control points on the humanoid robot head, and then we used a flexible VG Sensor to measure strain on both the human face and the silicone skin of the humanoid robot head. A method was proposed to evaluate the degree of anthropomorphism in the robot’s facial expressions using the difference rate of maximum strain. The feasibility of this method was confirmed through experiments concerning facial expression recognition. Finally, the impulse was calculated based on the strain curves to compare the humanoid robot head’s energy consumption levels when completing different facial expressions. These data are then used to reflect the energy consumption of the human face in different emotional states from a physical perspective. Simultaneously, an impulse is used to reflect the energy consumption of each moving part, which provides valuable data for optimizing the mechanism and selecting drive components for the humanoid robot head.

## 2. Mechanical Design and Facial Expression Realization of the Humanoid Robot Head

### 2.1. Design and Manufacturing Process for a Humanoid Robot Head

**Figure 1:**
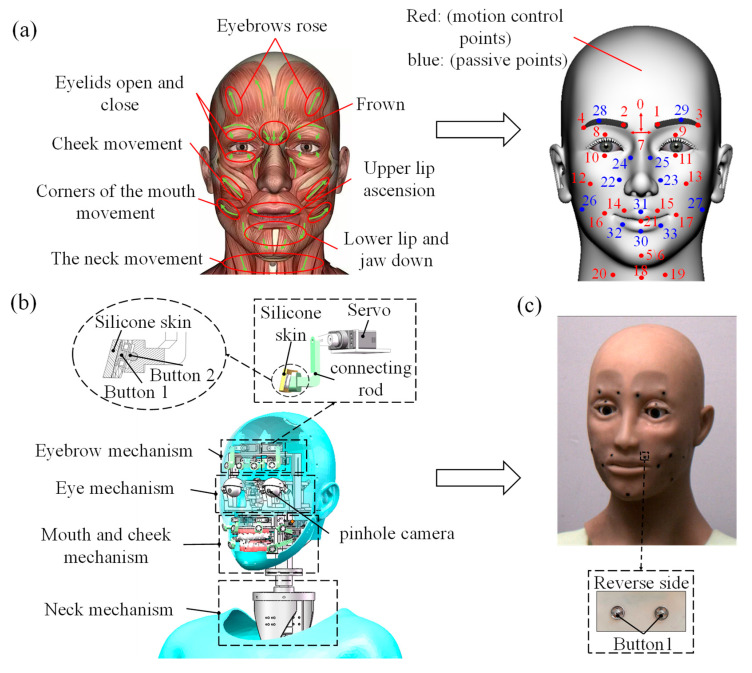
(**a**) Designation of facial movement control points. (**b**) Humanoid Robot Head Mechanisms and Power Output Mechanisms. (**c**) The humanoid robot head and snap button 1.

The human face is an intricate structure that comprises 44 muscles [32]. In Figure 1a, based on human facial muscle movements and the Facial Action Coding System (FACS) [33], we have designated 32 drive points to elucidate the movement of 21 Action Units (AUs) that humans use to express facial emotions. The 19 red points in Figure 1a [34] represent motion control points on the silicone skin of the humanoid head robot, whereas the blue points indicate passive points. As shown in Figure 1b, the humanoid robot head was designed with 19 degrees of freedom, as follows: 4 for the eyebrow mechanism, 4 for the eye mechanism, 8 for the cheek and mouth mechanism, and 3 for the neck mechanism. The mechanism design utilizes a servo motor for the drive, with the driving force directly transmitted to the silicone skin through a rigid linkage and snap button connection. In this design, snap button 1 is connected to the silicone skin, and snap button 2 is connected to the rigid linkage through a ball-and-socket joint. The facial skin of the humanoid robot head was manufactured using simulated silicone skin, and in Figure 1c, the humanoid robot head prototype is shown, as is snap button 1, which is connected to the motion control point on the opposite side of the silicone skin.

### 2.2. The Facial Expression Realization of the Humanoid Robot Head

**Figure 2:**
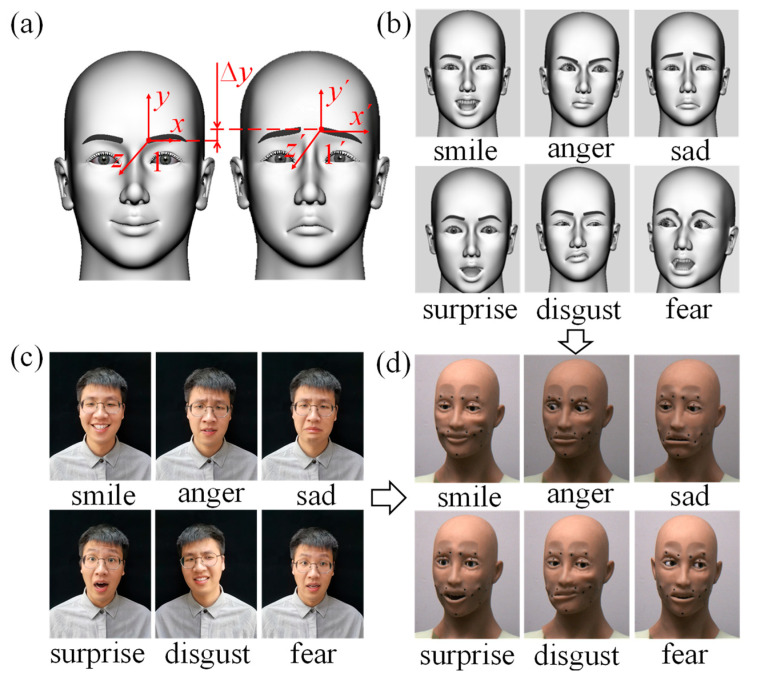
(**a**) Example of an established 3D coordinate system for control points in the humanoid head model. (**b**) The simulation of the six basic facial expressions. (**c**) The six basic facial expressions of humans. (**d**). The six basic facial expressions of the humanoid robot head.

Basic facial expression simulation was achieved by setting 19 identical motion-driven points in the humanoid head model, as depicted in Figure 1a, in Section 2.1 of this paper. Then, we used AdvancedSkeleton to add controllers to these points, in order to enable facial expression simulation in the humanoid head model. The movement displacements of the different parts were determined based on the research conducted by Ishihara et al. [35], who tracked the three-dimensional positions of 125 Japanese male faces. Furthermore, when simulating the six basic facial expressions, the simulation was carried out with the aim of achieving the maximum intensity of each facial expression. A 3D coordinate system was established at each motion control point of the humanoid head model, as exemplified by control point 1 in Figure 2a. We analyzed the relative displacement of each motion control point while simulating facial expressions in the humanoid head model. Then, we established a connection between the facial expressions and muscle displacement parameters to guide the parameter settings in the humanoid robot head’s control system. The motion parameters for the drive points corresponded with different parts of the six basic facial expressions, which are detailed in Table A1 of Appendix A.

The motion parameters of each drive point were optimized based on the amplitude of facial muscle movements, which corresponded with the six basic facial expressions of the human face, as shown in Figure 2c. Subsequently, controllers in the simulation software were used to provide the corresponding displacements of the control points, which completed the simulation of the six basic facial expressions, as illustrated in Figure 2b [36].

The realization of facial expressions in the humanoid robot head occurred when the control system of the humanoid robot head used a Raspberry Pi as the master controller, which communicated with a servo control board through a serial port to control the movements of the servos. The servo control parameters of the humanoid robot head were then set in accordance with the motion parameters of the drive points in Table A1, of Appendix A. The control system of the humanoid robot head was then programmed to achieve six basic facial expressions, as shown in Figure 2d.

## 3. Experiments

### 3.1. Facial Expression Recognition Experiments and Results

To evaluate whether the facial expressions achieved by the humanoid robot head were recognized, we presented images of the robot expressing six basic facial expressions to 210 participants (untrained individuals aged between 10 and 70 years), and we required them to match one of the six facial expressions to the images. The experiment produced recognition results, as depicted in Figure 1, where the diagonal section indicates the number of correctly recognized individuals. As per Table 1, the probability of correctly recognizing all six basic facial expressions that can be achieved by the humanoid robot head is over 80%, thus indicating a high degree of anthropomorphism for the six basic facial expressions that can be achieved by the humanoid robot head in this study. The recognition rate for the sad expression is highest at 97.14%, whereas the recognition rate for the angry expression is lowest at 80.48%. The ranking of the recognition rates for the six basic facial expressions is as follows: sad > smile > disgust > surprise > fear > anger.

### 3.2. Experimental Design of Strain Measurement

The humanoid robot head expresses emotions through the deformation of its silicone skin, making it necessary to study the strain on the silicone skin during the facial expression process. More specifically, comparing the strain on the same moving part of the human face and humanoid robot head provides valuable data references for improving the anthropomorphism of the robot’s facial expressions. In this experiment, we used a flexible VG Sensor to measure strain on both the human face and the silicone skin of the humanoid robot head. The dimensions of the VG Sensor in the experiment were length × width × height (60 mm × 8 mm × 0.8 mm). The average thickness of the silicone skin was 2 mm. The calibration established a functional relationship between the strain of the VG Sensor and the voltage at its ends, which may be represented as $y_{i}=ax_{i}$. The calibration results for each VG Sensor were detailed in Table A2, in Appendix A.

**Figure 3:**
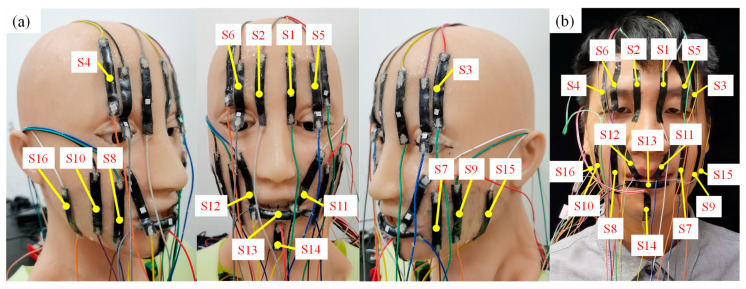
(**a**) The measurement site and number of VG Sensors on the silicone skin; (**b**) the measurement site and number of VG Sensors on the human face.

During the experimental design for strain measurement, the measurement site for the VG Sensor was determined based on the distribution of the red motion control points in Figure 1a and the facial expressions in Figure 2b,c. Identical strain measurement sites were established on both the silicone skin of the humanoid robot head and the human face (taking one of the participants as an example, and with their consent). As illustrated in Figure 3a,b, the 16 VG Sensors used for experimental measurements were labelled with numbers. The VG Sensors were fitted closely to the surface of the silicone skin and the human facial skin in the experiment, and the strain was measured by equally deforming the silicone skin and the human facial skin. During the experiment, the strain on the silicone skin was measured as the humanoid robot head completed six basic facial expressions, with three different action transition times of 100 ms, 500 ms, and 3000 ms. Notably, the eyelid area of the humanoid robot head completed movements with an action transition time of 100 ms. The motion control parameters of the humanoid robot head were set, based on the relevant parameters, which are shown in Table A1 of Appendix A. The movement parameters for the different parts of each facial expression in Table A1 were derived from the simulation results in Section 2.2. And the six basic facial expressions were simulated to achieve the maximum degree of expression for each specific facial expression. The strain measurement experiment on the human face involved five participants. They were instructed to mimic the intensity of six basic facial expressions, as shown in Figure 2b. Each participant completed each facial expression at least five times, and the strain values from various facial regions were measured while completing each facial expression.

In the experiment, the distribution and measurement range of VG Sensors were as follows: S1 and S2 were used to measure the strain on the left and right eyebrows; S3 and S4 were used to measure the strain on the left and right tips of the brow; S5 and S6 were used to measure the strain on the left and right eyelids; S7 and S8 were used to measure the strain on the left and right corners of the mouth; S9 and S10 were used to measure the stress on the left and right cheeks; S11 and S12 were used to measure the strain on the left and right upper lip; S13 was used to measure the strain on the lower lip; S14 was used to measure the strain on the jaw; and S15 and S16 were used to measure the strain on the left and right sides of the cheek.

### 3.3. Strain Measurement Results

**Figure 4:**
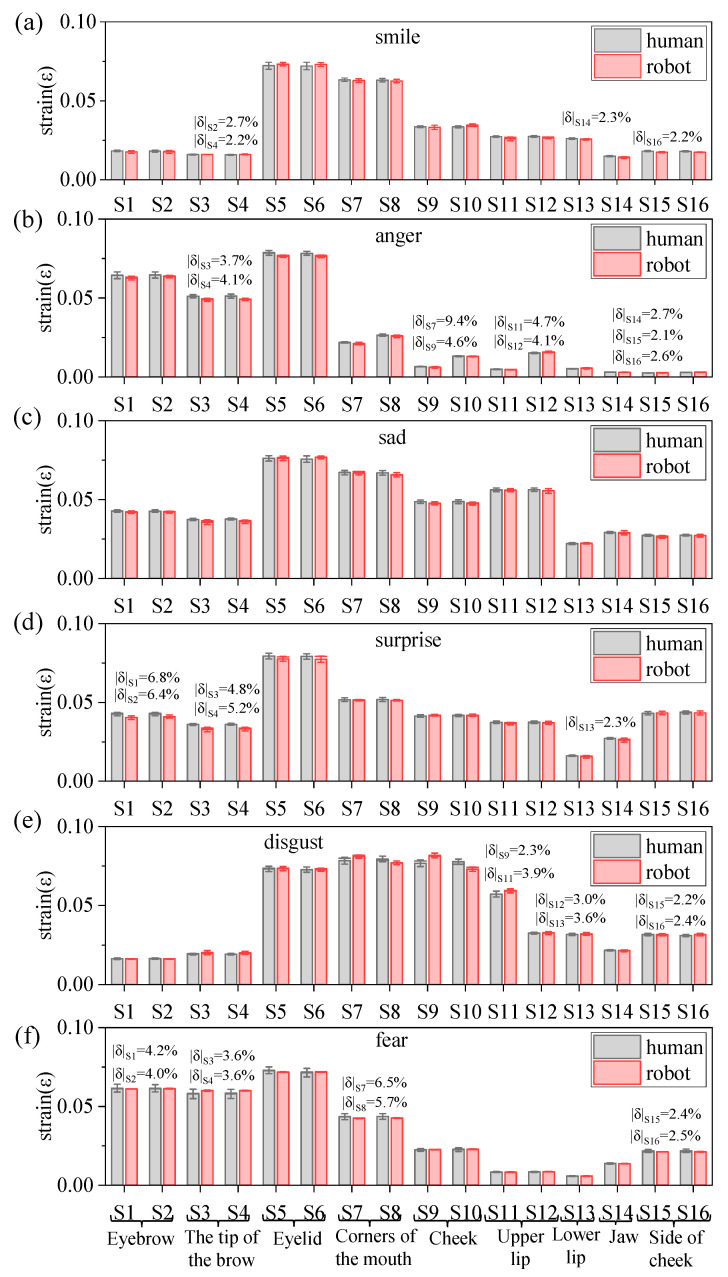
Comparison of the maximum strain values in different parts of the human face (based on the five participants) and the humanoid robot head when completing smiling (**a**), angry (**b**), sad (**c**), surprised (**d**), disgusted (**e**), and fearful (**f**) facial expressions.

The strain measurement results for the human face and silicone skin of a humanoid robot head were compared and analyzed. The maximum strain comparison diagram for each moving part of the human face for the five participants and the humanoid robot head, as smiling, angry, sad, surprised, disgusted, and fearful facial expressions were completed, is shown in Figure 4a–f.

Based on the experimental results, we calculated the difference between the rate of maximum strain on the moving parts of the human faces of the five participants and the rate of maximum strain on the same moving part of the humanoid robot head when completing an identical facial expression. We used the differential rate to evaluate the similarities between the humanoid robot head and the human face when completing identical facial expressions. The formula for calculating the differential rate is as follows:

(1) $$\delta =\frac{\varepsilon_{r}-\varepsilon_{h}}{\varepsilon_{h}}$$

where, δ is the difference rate of the maximum strain; $\varepsilon_{h}$ is the average of the maximum strain values for each part of the human face (based on the faces of the five participants); $\varepsilon_{r}$ is the maximum strain value for each part of the humanoid robot head.

Silicone skin samples of length × width × height (60 mm × 8 mm × 2 mm) were fabricated in accordance with the dimensions of the upright graphene used in the experiments, and they were subjected to tensile tests at different lengths. Referring to the maximum displacement in Table A1 of Appendix A, and stretching the silicone skin sample by 10 mm, the calculated strain value in this condition was 0.167. Moreover, several silicone skin samples with identical dimensions were elongated by 10.25 mm, 10.5 mm, 10.75 mm, 11 mm, 11.25 mm, 11.5 mm, 11.75 mm, and 12 mm, respectively. Subsequently, one hundred participants, unaware of the stretching conditions, were asked to compare these elongated silicone skin samples of variable lengths with those stretched by 10 mm. Statistical data indicate that at a stretch length of 10.25 mm, the probability of equivalence for a stretch length of 10 mm peaks at 97.28%, whereas at 10.5 mm, this probability reduces to 72.32%. Moreover, as the stretch length increases, the probability of equivalence for a stretch length of 10 mm decreases. The calculated difference rate between stretch lengths of 10 mm and 10.25 mm is 2.29%. Therefore, this paper specifies an acceptable range for the difference rate δ, which is $\left[-2\%,2\%\right]$. More specifically, when the difference rate δ at a specific measurement site is within the $\left[-2\%,2\%\right]$ interval, it is considered that the motion of the humanoid robot head and the human face (based on the faces of the five participants) at this measurement site is consistent. As shown in Figure 4, the average values of $\left|\delta \right|$ which exceeded the acceptable range, are indicated in the corresponding strain bars on the graph.

Firstly, we compared the amplitude of movement in the symmetrical parts of the left and right-hand sides of the faces using the δ. The results show that the δ for the symmetrical parts of the left and right-hand sides of the faces when completing the smiling, sad, surprised, and fearful facial expressions are all within the acceptable range. This indicates that the amplitude of movement in the left and right-hand sides of the faces, when completing these four facial expressions, is approximately equal.

The δ values for the measurement sites of VG Sensors S7 and S8, S9 and S10, S11 and S12, and S15 and S16, when completing the anger and disgust facial expressions, fell outside of the acceptable range. This indicates that the amplitude of movement at the left and right corners of the mouth, the left and right cheeks, the left and right upper lips, and the left and right cheek sites was asymmetrical when completing the anger and disgust facial expressions. More specifically, when completing the angry and disgusted facial expressions, the humanoid robot head exhibits low symmetry in the left and right upper lip areas, with differential rates of −70.97% and 77.86%, respectively.

Secondly, the δ was used to evaluate the similarity between the humanoid robot head and the human face when completing the same facial expression. The larger the absolute value of the differential rate of the maximum strain ($\left|\delta \right|$) in a specific measurement site, the lower degree of similarity in that part. According to Figure 4a, the smiling facial expression exhibits four areas where the δ value falls outside the acceptable range, with the largest value of $\left|\delta \right|$ being 2.7% in the right eyebrow tip area. According to Figure 4b, the angry facial expression exhibits nine areas where the δ value falls outside the acceptable range, with the largest value of $\left|\delta \right|$ being 9.4% in the left corners of the mouth, which numerically exceeds the acceptable range by a significant margin. This indicates a low degree of similarity between the angry facial expression completed by the humanoid robot head and that completed by the human face. However, as seen in Figure 4c, the δ values for all parts of the sad facial expression are within the acceptable range, which indicates a high degree of similarity between the sad expression completed by the humanoid robot head and the human face. As shown in Figure 4d, the surprised facial expression exhibits five areas where the δ value falls outside the acceptable range, mainly in the eyebrow and tip of the eyebrow areas, with the largest value of $\left|\delta \right|$ being 6.8% in the left eyebrow area. This result indicates that there was a low degree of similarity between the surprised facial expression completed by the humanoid robot head and that completed by the human face due to the differences between the eyebrow and the tip of the eyebrow when the humanoid robot head and the human face were producing this expression. Figure 4e highlights that the disgusted facial expression exhibits six areas where the δ value falls outside the acceptable range, mainly in the mouth and cheek areas, with the largest value of $\left|\delta \right|$ being 3.9% in the left upper lip area. As shown in Figure 4f, the fearful facial expression comprises eight parts where the δ value falls outside the acceptable range, the largest value of $\left|\delta \right|$ being 6.5% in the left corners of the mouth. In summary, for parts with low similarity, the motion optimization suggestions, based on their δ values, are as follows: when δ exceeds 2%, reduce the displacement output of the mechanism in proportion to δ, and when δ is less than −2%, increase the displacement output of the mechanism so that it is inversely proportional to δ.

**Figure 5:**
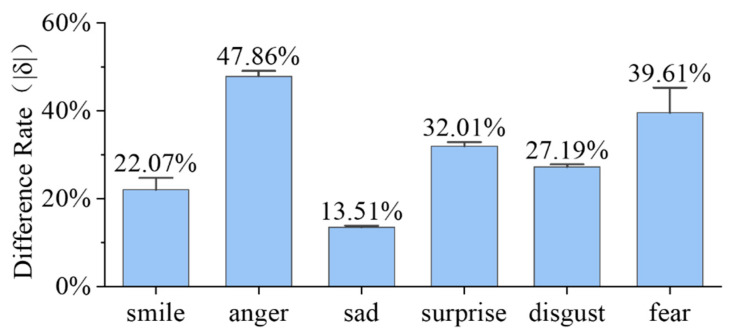
The sum of the absolute value of the differential rate of the maximum strain ($\left|\delta \right|$) for all measurement sites of the humanoid robot head when completing the different facial expressions.

Figure 5 illustrates the comparison between the sum $\left|\delta \right|$ of all measurement sites on the face when completing facial expressions. By analyzing Figure 5, we can obtain the similarity ranking of the six basic facial expressions completed by the humanoid robot head and the six basic facial expressions completed by the human face. It is as follows: sad > smile > disgust > surprise > fear > anger. Its ranking matches that of the recognition rates of the six basic facial expressions in facial expression recognition experiments. This indicates the feasibility of evaluating the anthropomorphism of humanoid robot head facial expressions using strain difference rates.

In conclusion, we proposed a method to evaluate the degree of anthropomorphism in a robot’s facial expressions based on the experimental results. This was achieved by using the difference rate of the maximum strain values on the same parts. Simultaneously, we validated the feasibility of the proposed robot facial expression evaluation method through a comparison of the results of the facial expression recognition experiments. The evaluation indicates that there are differences between certain regions in the humanoid robot head and the human face when completing the same facial expression. Nevertheless, these differences do not significantly impact the recognition of the overall facial expression. Hence, the six basic facial expressions completed by the humanoid robot head and the human face are highly similar to each other.

Finally, the impulse for each part of the humanoid robot head when making a facial expression was used to describe the magnitude of energy consumption. The impulse for each part of the humanoid robot head when completing a facial expression was calculated based on the strain curve measured in the experiment, and the calculation method is given in Equation (2) as follows:

(2) $$I_{i}=\int_{t_{0}}^{t_{1}}F_{\mathrm{SIL}}dt$$

where: $I_{i}$ is impulse; $F_{\mathrm{SIL}}$ is the function of the force on the silicone skin; $t_{0}$ is the initial moment when the humanoid robot head completes the facial expression; $t_{1}$ is the moment after the humanoid robot head completes the facial expression. 

**Figure 6:**
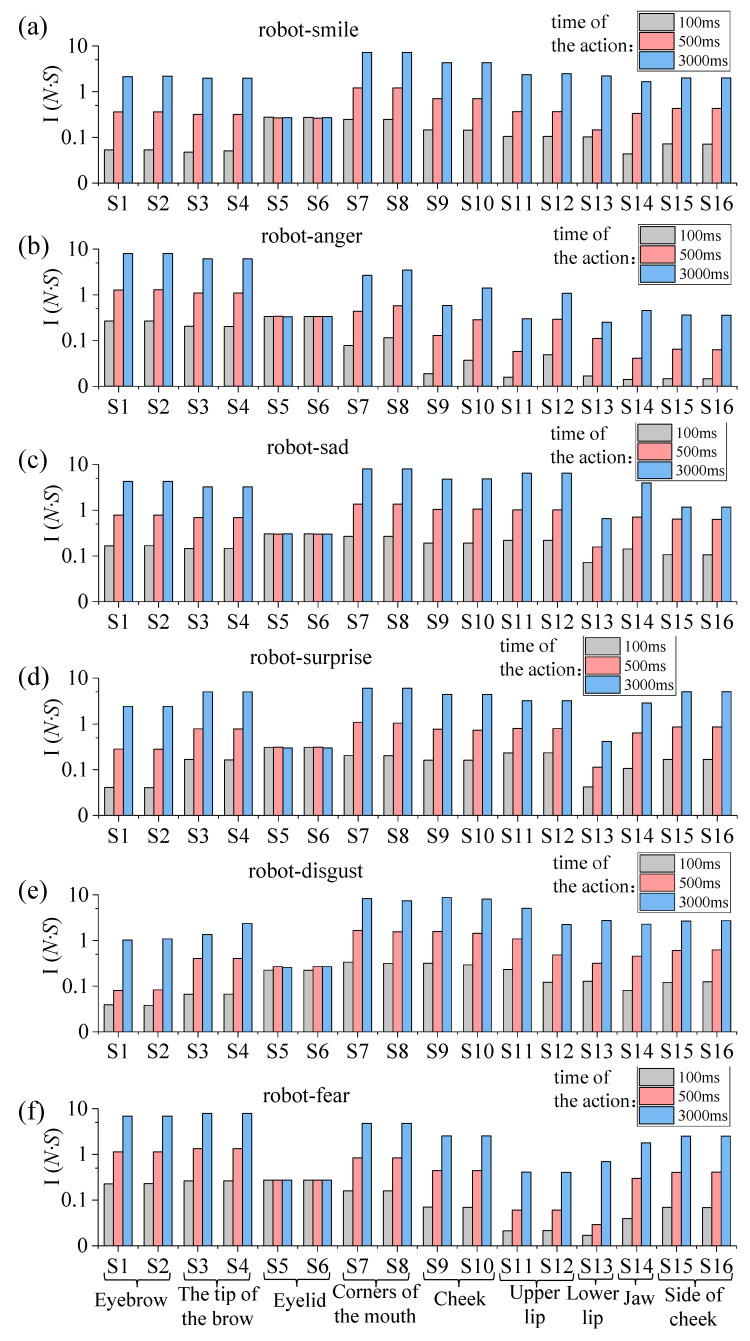
Impulses for each part of the humanoid robot head when it completes the following facial expressions: smiling (**a**), anger (**b**), sadness (**c**), surprise (**d**), disgust (**e**), and fear (**f**).

The impulses for each part of the humanoid robot head were calculated while completing six basic facial expressions, with three action transition times of 100 ms, 500 ms and 3000 ms, as shown in Figure 6.

As shown in Figure 6, the impulse value of the moving parts of the humanoid head robot increases proportionally with the action transition time when completing the same facial expression. Additionally, when the humanoid robot head completes a facial expression with a 100 ms action transition time, it closely resembles the time required for a human face to complete the same expression.

After analysing the impulse values of each part of the humanoid robot head when completing the six basic facial expressions with the same action transition time, the impulse values of the left and right eyebrow parts are ranked as: *I_anger_ > I_fear_ > I_sad_ > I_smlie_ > I_surprise_ > I_disgust_*; the impulse values of the left and right tip of brow parts are ranked as: *I_fear_ > I_anger_ > I_surprise_ > I_sad_ > I_disgust_ > I_smlie_*; the impulse values of the left and right eyelid parts are ranked as: *I_anger_ > I_surprise_ > I_sad_ > I_smlie_ > I_fear_ > I_disgust_*; the impulse values of the left and right corners of the mouth parts are ranked as: *I_disgust_ > I_sad_ > I_smlie_ > I_surprise_ > I_fear_ > I_anger_*; the impulse values of the left and right cheek parts are ranked as: *I_disgust_ > I_sad_ > I_surprise_ > I_smlie_ > I_fear_ > I_anger_*; the impulse values of the left upper lip parts are ranked as: *I_surprise_ > I_disgust_ > I_sad_ > I_smlie_ > I_fear_ > I_anger_*; the impulse values of the right upper lip parts are ranked as: *I_surprise_ > I_sad_ > I_disgust_ > I_smlie_ > I_anger_ > I_fear_*; the impulse values of the jaw parts are ranked as: *I_sad_ > I_surprise_ > I_disgust_ > I_smlie_ > I_fear_ > I_anger_*; the impulse values of the left and right sides of the cheek are ranked as: *I_surprise_ > I_disgust_ > I_sad_ > I_smlie_ > I_fear_ > I_anger_*. The impulse values of different parts of the humanoid robot head when completing facial expressions with a 100 ms action transition time were analyzed in order to assess its energy consumption. In general, parts with larger motion amplitudes consume more energy. Furthermore, based on the magnitude of energy consumption, this provides data support for the selection of driving components for various parts. It is evident that when the humanoid robot completes the six facial expressions, the energy consumption of the eyelids and corners of the mouth is relatively higher than other parts. Therefore, it is necessary to select servos with a larger torque for the eyelids and corners of the mouth in order to meet the energy demand for these areas of movement.

**Figure 7:**
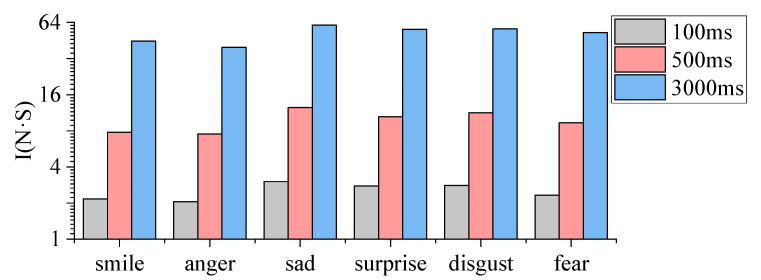
The sum of impulse values corresponding with different facial expressions.

Normally, when the facial expression recognition rate exceeds 80%, it indicates that humans can easily recognize facial expressions, indicating a high level of facial expression replication. During the experiment, the humanoid robot head achieved six basic facial expressions based on the maximum degree of each expression, and the results show that the recognition rate of the completed facial expressions exceeded 80%. This study assumed that the energy consumption of the humanoid robot, to generate facial expressions with a recognition rate that exceeds 80%, could serve as an approximation for the energy required to complete these expressions. Hence, we calculated the sum of impulse values for all parts of the humanoid robot head when completing a specific facial expression to reflect the energy consumption for that facial expression. Figure 7 illustrates the sum of impulse values corresponding with different facial expressions.

From Figure 7, it is evident that the sum of impulse values increases as the time of the action transition increases when the humanoid robot head completes a facial expression. This also indicates that the more time taken to complete a facial expression, the greater the energy consumption. Additionally, under the same action transition time conditions, the ranking of energy consumption for the humanoid robot head when completing the six basic facial expressions is as follows: sad > disgust > surprise > fear > smile > anger.

## 4. Discussion

Improving the operational efficiency and lifespan of the humanoid robot head holds great significance. In the humanoid robot head designed in this study, the driving force behind the mechanism is efficiently transmitted to the silicone skin through a rigid linkage drive and snap button connection to achieve a facial expression. Compared with the pneumatic bionic muscle drive [37], the use of a rigid linkage to transmit the driving force to the silicone skin enhances the response speed and load capacity. Compared with the rope drive [38], the use of a rigid linkage to transmit the driving force to the silicone skin avoids a phenomenon where the silicone skin relaxes and it improves the movement precision and transmission efficiency. Secondly, the silicone skin is connected to the movement mechanism with a snap button. This connection facilitates the installation of the silicone skin when debugging the mechanism, and it increases the lifespan of the silicone skin compared with gluing it to the mechanism drive point [39]. 

Currently, the evaluation of the degree of anthropomorphism in robot facial expressions is mainly achieved by obtaining the recognition rate through subject recognition experiments [40,41]. This evaluation method effectively measures the realism of the facial expressions achieved by the humanoid robot head. Normally, when the facial expression recognition rate exceeds 80%, it indicates that humans can easily recognize facial expressions, indicating a high level of facial expression replication. In this study, 210 subjects were recruited to perform expression recognition tasks for the facial expressions achieved by the humanoid robot head. The results revealed a correct recognition rate of over 80% for the six basic facial expressions achieved by the humanoid robot head, indicating a high level of anthropomorphism in the facial expressions achieved by the designed humanoid robot head. The recognition rate for the sad expression was the highest at 97.14%, while the recognition rate for the angry expression was the lowest at 80.48%. The ranking of the recognition rates for the six basic facial expressions is as follows: sad > smile > disgust > surprise > fear > anger.

Additionally, Almayman et al. [42] and Hsu et al. [43] conducted pioneering research on the strain distribution of human facial expressions; however, their primary focus was on the strain associated with specific facial movements. On this basis, this paper experimentally measured the strain in each part of the face, and it evaluated the degree of anthropomorphism in the robot’s facial expression in terms of physical quantities. Surface strain measurement on the human face and silicone skin is a challenging problem. However, in this study, a new flexible VG Sensor was used. These sensors can fit closely to the surface of the human face and silicone skin, producing equal levels of deformation to enable the measurement of strain on the human face and the moving parts of the humanoid robot head when completing facial expressions. Experimental measurements indicated that the highest strain in the human facial region was observed in the eyelid area, followed by the corner of the mouth. This is consistent with the findings of Misu et al.’s research on strain in the human face, which also emphasised significant strain in the eye and mouth regions. In this experiment, the maximum strain value measured in the human facial eyelid region was 0.082, which differs numerically from the findings of Misu et al.’s study, where the maximum strain was approximately $E_{\mathrm{area}}>0.2$. Although Misu et al. established a reference range for strain in different facial regions, this study directly measured the specific strain values for various facial regions during the performance of facial expressions by humans. These detailed data references can effectively assist other researchers.

Furthermore, this study proposes a method to quantitatively evaluate the degree of anthropomorphism in robot facial expressions by using the difference rate of the maximum strain values of the same parts of the humanoid head and human face. Five participants were invited to attempt to replicate the intensity of the six basic facial expressions depicted in Figure 2b. Simultaneously, the strain values of various facial regions were measured each time a facial expression was completed by the participants. Although each emotion can be expressed to various extents, in this study, both the humanoid robot head and the five participants replicated the intensity of the six basic facial expressions based on the simulation results. In addition, it was assumed that the intensity of the facial expressions completed by both the humanoid robot head and the participants is identical. Thus, comparing the strains of the various facial regions of the humanoid robot head and human faces, under conditions of equal expression intensity, is deemed acceptable. Then, the difference rate was calculated for the average maximum strain values of various facial regions, when the five participants completed the same expression, and the maximum strain values of the corresponding facial regions when the humanoid robot achieved the same facial expression. Subsequently, the absolute sum of the difference rates of the maximum strain values for all facial regions of the humanoid robot head and the five participants who completed the six basic facial expressions was determined, resulting in a ranking denoted as: sad > smile > disgust > surprise > fear > anger. Its ranking matches that of the recognition rates of the six basic facial expressions in facial expression recognition experiments. This indicates the feasibility of evaluating the anthropomorphism of humanoid robot head facial expressions using strain difference rates. Few studies have focused on the optimization of the structure of the humanoid robot head. However, this study presents optimization guidelines for motions and mechanisms based on the δ value of the moving parts when completing facial expressions. This contributes to improving the anthropomorphism of the humanoid robot head’s facial expressions. 

The humanoid robot head designed in this study exhibited a relatively high degree of anthropomorphism in terms of its ability to achieve the six basic facial expressions. However, the reproduction of different facial expressions by the humanoid robot head varies. Table A1 in Appendix A shows the number of motion control points required to achieve the six basic facial expressions: sadness (eight control points), smile (eight control points), disgust (nine control points), surprise (ten control points), fear (thirteen control points), and anger (fourteen control points). These data were integrated into an analysis concerning the ranking of anthropomorphism levels in the humanoid robot head and its ability to execute these expressions. It is evident that as the number of motion control points needed to achieve facial expressions increases, reproducing these facial expressions becomes more challenging. Consequently, this difficulty results in the facial expressions accomplished by the humanoid robot head experiencing a diminished level of fidelity. When the number of motion control points required to achieve facial expressions is consistent, the analysis focused on the magnitude of the motion displacement of these points. Greater motion displacement makes it easier for humans to discern differences, facilitating the reproduction of facial expressions. The increased visibility of differences contributes to the easier reproduction of facial expressions.

In the experiment, the humanoid robot head achieved six basic facial expressions, based on the maximum degree of each facial expression, and the results show that the recognition rate of the achieved facial expressions exceeded 80%. This study assumed that the energy consumption of the humanoid robot head, to generate facial expressions with a recognition rate that exceeds 80%, could serve as an approximation of the energy required to complete these expressions. Hence, we calculated the sum of impulse values for all parts of the humanoid robot head when completing a specific facial expression, which was used to reflect the energy consumption of that facial expression. Under the same action transition time conditions, we obtained the ranking for the energy consumption of the humanoid robot head when achieving the six basic facial expressions. It is as follows: sad > disgust > surprise > fear > smile > anger. Certainly, the energy consumed by the humanoid robot head decreases as the intensity of the expression decreases. However, in this study, each facial expression was executed at its maximum intensity. Thus, the research findings still offer valuable insights. In this study, based on the experimental measurement results, we calculated the impulse values of different parts when the humanoid robot head completed expressions. These impulse values were used to reflect the energy consumption of each part, offering data support for the selection of the drive components. Although the appearance and skin material of the humanoid robot heads that were designed by different researchers may vary, these differences are not essential disparities. Hence, they exhibit similarities with the humanoid robot designed in this study, which aimed to mimic human features. When different materials are used to fabricate the facial skin of humanoid robots, each part of the humanoid robot’s skin is typically made of a uniform material. In studies involving humanoid robot head facial skins with diverse materials, researchers can first measure the force required for a specific part of the humanoid robot’s face to perform an action, and then select appropriate actuators accordingly. Subsequently, utilizing the energy consumption ratio of different parts of the humanoid robot head, as described in this study, the researchers quickly selected actuators for different head regions, thus reducing the design time of the humanoid robot head. Furthermore, the judicious selection of actuators for each part can significantly reduce the manufacturing cost of the humanoid robot head. The movement of the different parts of humanoid robot heads with varying appearances when achieving facial expressions resembles that of the humanoid robot head in this study. Throughout the design process, researchers can also refer to relevant data from this study. In essence, the energy consumption patterns of different parts of the humanoid robot head, as outlined in this research, can serve as valuable data references for other researchers involved in humanoid robot head design. Describing the energy consumption of the human face in different emotional states is a challenging problem. Assuming that the skin of various parts of the human face is uniform, and that the energy consumed by human facial skin is directly proportional to the displacement produced, the energy consumption of the humanoid robot head when achieving different facial expressions, as depicted in this study, can provide insights into the energy consumption of the human face when achieving different facial expressions.

The humanoid robot head designed in this study successfully achieved six basic facial expressions. The evaluation, using the strain difference rate and facial expression recognition experiments, illustrates that the overall anthropomorphic level of the facial expressions achieved is high. Nevertheless, there are areas where certain facial expressions show insufficient similarities, such as the eye area for the “surprise” facial expression, the corners of the mouth and cheek areas for the “disgust” facial expression, and the mouth, cheek, and jaw areas for the “anger” facial expression. Regarding future research on humanoid robots, the focus will be on the intelligence of robots [44], with the realism and richness of facial expressions being the most crucial factors influencing their intelligence. However, the research content of this paper is of significant importance for enhancing the realism of facial expressions in humanoid robots.

## 5. Conclusions

In this study, a humanoid robot head was designed, in which the driving force of the mechanism was efficiently transmitted to the silicone skin through a rigid linkage drive and snap button connection. Facial expression recognition experiments were conducted to obtain the recognition rate ranking of the six basic facial expressions of the humanoid robot head. Then, a new flexible VG Sensor was used to measure the strain on both the human face and the silicone skin of the humanoid robot head when completing facial expressions. Next, based on the measurement results, a method was proposed to evaluate the degree of anthropomorphism in the robot’s facial expressions by using the difference rate of the maximum strain value in the same area. And the impulse value was used to reflect the energy consumption of the humanoid robot head when completing facial expressions. The main conclusions that can be drawn from this work are as follows:

(1) The rigid linkage drive design improves the response speed and load carrying capacity, and it solves the problem of low motion accuracy and drive efficiency caused by the silicone skin relaxation phenomenon. The snap button connection between the silicone skin and the drive link increases the lifespan of the silicone skin.
(2) The consistency between the results of the evaluation of the robot’s facial expressions, using the strain difference rate and facial expression recognition rate, validates the feasibility of the proposed method. The anthropomorphism ranking for the six basic facial expressions of humanoid robot head is as follows: sad > smile > disgust > surprise > fear > anger. This was based on the δ value of each part, which provided a data reference for its mechanism and motion optimisation.
(3) As the number of control points required to replicate facial expressions increases and the movement displacement of these control points decreases, reproducing these facial expressions becomes more challenging.
(4) The energy consumption of each part is reflected by its impulse value, providing data to support the selection of drive components for other researchers. It has been established that the humanoid robot head consumes more energy in the eyelids and corners of the mouth when completing facial expressions. Therefore, a servo with a larger torque should be selected to meet the energy demand of these two moving parts.
(5) When the facial expression recognition rate exceeds 80%, the ranking for the energy consumption of the humanoid robot head when completing the six basic facial expressions is: sad > disgust > surprise > fear > smile > anger.

In conclusion, this paper designed a humanoid robot head capable of achieving six basic facial expressions with a high degree of anthropomorphism. Additionally, it proposed an evaluation method for the degree of anthropomorphism for robot facial expressions based on the difference rate of the maximum strain. The feasibility of this method was verified. However, further research is needed to develop more intelligent humanoid robot heads.

## Figures and Tables

**Table 1:** Facial expression recognition results for the humanoid robot head.

		Options						Recognition Rate
		Smile	Anger	Sad	Surprise	Disgust	Fear	
images	Smile	200	3	1	3	1	2	95.24%
	Anger	3	169	8	6	6	18	80.48%
	Sad	0	1	204	0	2	3	97.14%
	Surprise	3	6	2	182	1	16	86.67%
	Disgust	0	5	4	2	190	9	90.48%
	Fear	0	7	1	23	3	176	83.81%

## Data Availability

The data generated and/or analyzed during the current study are not publicly available for legal/ethical reasons but are available from the corresponding author up-on reasonable request.

## Appendix A

**Table A1:** Motion parameters of drive points for different parts of the six basic facial expression (cm).

	Motor Area	The Displacement of the X-Axis	The Displacement of the Y-Axis	The Displacement of the Z-Axis
Smile	Corners of the mouth (16, 17)	0~0.2	0~0.4	0~−0.02
	Upper lip (14, 15, 31)	0~0.04	−0.1~0.28	0~0.01
	Lower lip (30)	--	0~−0.32	--
	Jaw (5, 6)	--	0~−0.5	−0.05~0
Anger	Eyebrow center (3, 4)	0~−0.01	0~−0.30	--
	eyebrow (1, 2)	0~0.07	0~−0.35	--
	Lower lip (30)	0~−0.25	0~−0.15	0~0.2
	eyelid (8, 9, 10, 11)	0~0.01	0~0.05	--
	Upper lip (14, 15, 31)	0~0.01	0~0.31	--
	Jaw (5, 6)	--	0~0.05	--
Sad	eyebrow (1, 2)	0~−0.06	0–0.95	--
	Corners of the mouth (16, 17)	0~0.2	0~−0.6	--
	Upper lip (31)	--	--	0~0.2
	Lower lip (30)	--	0~0.02	0~0.2
	eyelid (8, 9)	0~0.15	--	--
Surprise	Eyebrow center (3, 4)	--	0~0.50	--
	eyebrow (1, 2)	--	0~0.27	--
	Jaw (5, 6)	--	0~−1.05	0~−0.3
	eyelid (8, 9, 10, 11)	--	0~−0.20	--
Disgust	Upper lip (15)	0~0.16	0~0.65	0~0.04
	Lower lip (5, 6)	--	0~0.4	--
	Corners of the mouth (16, 17)	0~0.3	0~−0.21	--
	eyelid (8, 9, 10, 11)	--	0~0.03	--
Fear	eyebrow (1, 2)	0~−0.10	0~0.12	--
	Eyebrow center (3, 4)	0~0.3	0~−0.69	0~0.09
	Upper lip (14, 15, 31)	--	0~0.40	--
	Jaw (5, 6)	0~0.03	0~−0.75	--
	eyelid (8, 9, 10, 11)	--	0~0.32	--

**Table A2:** Calibration results of 16 vertical graphene sensors.

**NO.**	Sensor 1 (S1)	Sensor 2 (S2)	Sensor 3 (S3)	Sensor 4 (S4)
calibration results	*y*_1_ = 3.176 × 10^−10^*x*_1_	*y*_2_ =1.073 × 10^−9^*x*_2_	*y*_3_ = 3.695 × 10^−10^*x*_3_	*y*_4_ = 4.151 × 10^−10^*x*_4_
**NO.**	Sensor 5 (S5)	Sensor 6 (S6)	Sensor 7 (S7)	Sensor 8 (S8)
calibration results	*y*_5_ = 3.559 × 10^−11^*x*_5_	*y*_6_ = 3.946 × 10^−11^*x*_6_	*y*_7_ = 8.405 × 10^−11^*x*_7_	*y*_8_ = 8.454 × 10^−11^*x*_8_
**NO.**	Sensor 9 (S9)	Sensor 10 (S10)	Sensor 11 (S11)	Sensor 12 (S12)
calibration results	*y*_9_ = 2.59 × 10^−10^*x*_9_	*y*_10_ = 7.655 × 10^−11^*x*_10_	*y*_11_ = 6.32 × 10^−9^*x*_11_	*y*_12_ = 6.169 × 10^−11^*x*_12_
**NO.**	Sensor 13 (S13)	Sensor 14 (S14)	Sensor 15 (S15)	Sensor 16 (S16)
calibration results	*y*_13_ = 3.45 × 10^−10^*x*_13_	*y*_14_ = 1.142 × 10^−9^*x*_14_	*y*_15_ = 2.2 × 10^−11^*x*_15_	*y*_16_ = 9.511 × 10^−11^*x*_16_

In the table, *y_i_* is the strain on the vertical graphene sensor *i*; *x_i_* is the voltage across the vertical graphene sensor.
